# Structure–Property Correlation in Phenyl N‑Substituted Imidazolium Protic Ionic Liquids

**DOI:** 10.1021/acs.jpcb.6c01792

**Published:** 2026-06-23

**Authors:** Nicole Abdou, Elisabet Ahlberg, Anna Martinelli

**Affiliations:** † Department of Chemistry and Chemical Engineering, 11248 Chalmers University of Technology, SE-412 96 Gothenburg, Sweden; ‡ Department of Chemistry and Molecular Biology, 3570 University of Gothenburg, SE-413 90 Gothenburg, Sweden

## Abstract

Establishing the correlation between chemical structure and physicochemical properties in protic ionic liquids is essential for their rationale design and application. In this context, the recently developed protic ionic liquids, benzylimidazolium bis­(trifluoromethylsulfonyl)­imide and *n*-phenylimidazolium bis­(trifluoromethylsulfonyl)­imide, are characterized with focus on phase behavior, thermal stability, intermolecular interactions, transport, and electrochemical properties, using a wide range of experimental methods. Compared to the chemical structure of the archetypal protic ionic liquid, 1-ethylimidazolium bis­(trifluoromethylsulfonyl)­imide, the addition of a phenyl group to the cation results in reduced thermal stability, increased viscosity, stronger intermolecular interactions, and lower ionic conductivity, while maintaining comparable fragility, and a wider electrochemical stability window.

## Graphical abstract

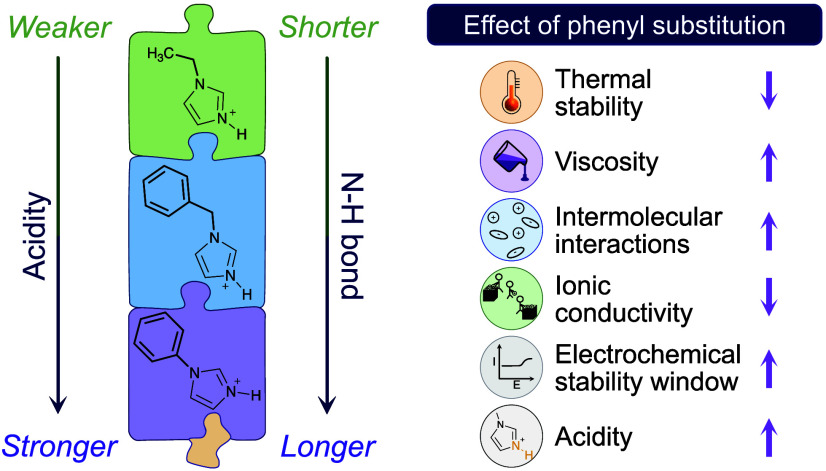

## Introduction

Ionic liquids (ILs), defined as molten salts with melting points below 100 °C, have attracted significant research interest due to their highly tunable physicochemical properties.^1,2^ Typically, ionic liquids exhibit high thermal and chemical stability, a broad electrochemical stability window, and good ionic conductivity.^3^ Among the various types of ionic liquids, protic ionic liquids are synthesized via a neutralization reaction between a Brønsted acid (proton donor) and a Brønsted base (proton acceptor).^2,4,5^ Although less extensively studied than their aprotic counterparts, protic ionic liquids have shown promising potential in a variety of applications, such as proton conductors in proton exchange membrane fuel cells,^6–8^ in homogeneous and heterogeneous catalysis,^9,10^ as well as electrolytes in Li-ion batteries.^11–13^

Various strategies have been used to tune the properties of ionic liquids, the most common being by varying the anion–cation pair^14^ and by modifying the structure of either ion, thus influencing acidity^15–17^ and charge delocalization.^18,19^

The tailoring of ionic liquid properties through structural modification has been explored for several decades. In 1992, Joan Fuller investigated modifications of dialkylimidazolium cations and examined various anion–cation combinations to improve stability in water.^20^ Later, Brzȩczek-Szafran et al. proposed enhancing the acidity of protic ionic liquids by synthesizing di- and triamino-based systems through proton transfer from sulfuric acid to the corresponding amines, aiming to improve and fine-tune their catalytic performance.^21^ Furthermore, Dahlqvist et al. reported increased acidity in imidazolium-based protic ionic liquids through the introduction of electron-withdrawing substituents on the cation, such as nitro and cyano substituents, thereby extending their potential for catalytic applications.^17^

Understanding and tuning the acidity of protic ionic liquids—i.e., modulating the strength of cation–anion interactions—therefore appears essential for optimizing their performance across different applications. For instance, acidity can significantly influence the catalytic activity of protic ionic liquids, as previously reported.^17,21^ It is also a key parameter in their use as electrolytes in lithium-ion batteries, where stronger cation–anion interactions may reduce ion coordination with lithium ions, potentially increasing Li^+^ mobility and improving electrolyte performance.^22^

Therefore, as an attempt to design and explore new electrolytes for Li-ion batteries based on protic ionic liquids, two protic ionic liquids based on the bis­(trifluoromethylsulfonyl)­imide [TFSI] anion and modified imidazolium cations, namely benzylimidazolium and n-phenylimidazolium, were systematically studied in this work. The benzyl- and phenyl- substituents on the imidazolium ring act as electron-withdrawing groups and may thus enhance the acidity of the exchangeable N–H proton, thereby influencing the strength of interactions between the imidazolium cation and the [TFSI] anion. The phase behavior, thermal stability, transport properties, intermolecular interactions, and electrochemical properties of these new protic ionic liquids are studied and compared to those of the more commonly used 1-ethylimidazolium bis­(trifluoromethylsulfonyl)­imide, contributing to a deeper understanding of the structure–property correlation in this class of materials.

## Experimental Section

### Materials

The protic ionic liquids 1-ethylimidazolium bis­(trifluoromethylsulfonyl)­imide (Y002x123.1-IL-0269), benzylimidazolium bis­(trifluoromethylsulfonyl)­imide (Y001x110.1-CS-2190), and *n*-phenylimidazolium bis­(trifloromethylsulfonyl)­imide (Y001x15.1-CS-2188), 98% purity, were purchased from Iolitec. All ionic liquids were stored in an N_2_ filled glovebox (MBRAUN UNIlab Plus Eco glovebox, equipped with an MB-LMF II solvent absorber system; ≤1 ppm of H_2_O; ≤1 ppm of O_2_) and used as received without further treatment. The molecular structures of the ions in these protic ionic liquids are given in Figure 1.

**Figure 1:**
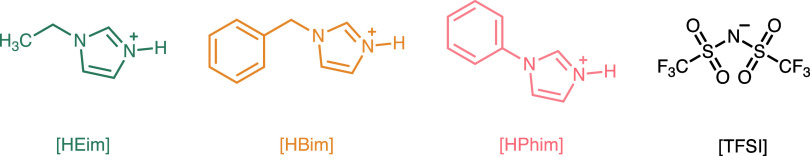
Molecular structure of the ions constituting the studied protic ionic liquids along with the acronyms used in the following text.

### Methods

#### Thermogravimetric Analysis (TGA)

TGA experiments were performed on a Mettler Toledo TGA/DSC3+, equipped with an autosampler. Approximately 20 mg of sample was weighed using a Mettler Toledo XS105 semi-micro balance and placed in a 70 μL aluminum crucible, which was capped by a lid with a pinhole. The samples were analyzed under air flow (60 μL/min) covering the temperature range 298–1073 K, with a heating rate of 10 K/min. The decomposition temperatures (*T*_d_) were determined from the peak minimum of the first derivative curve (Figure S1).

#### Differential Scanning Calorimetry (DSC)

DSC measurements were performed using a Mettler Toledo DSC5+ instrument equipped with an autosampler, a gas controller GC DT2 and a liquid nitrogen cooling system. 20 mg of sample was weighted and placed in 40 μL hermetic aluminum pans prior to measurement. DSC data were collected in the temperature range 150–353 K, using a cooling rate of 20 K/min and a heating rate of 10 K/min, under nitrogen flow. Three scans were recorded for each measurement. The glass transition temperature (*T*_g_) was determined from the second heating scan curve by identifying the inflection point of the fitting sigmoidal function.

#### Density and Viscosity

Density and viscosity values were collected using an Anton Paar DMA 4500 M oscillating U-tube densitometer over the temperature range 293–343 K, with 10 K increments. The density accuracy is about ±0.0001 g/cm^3^. The viscosity was determined using the Lovis 2000 ME rolling ball viscometer module. The viscosity measurement has an accuracy of up to 0.1% and a repeatability of 0.5%. The temperature accuracy is ± 0.2 K with a repeatability of ±0.005 K.

#### Impedance Spectroscopy

Conductivity measurements were carried out using a Novocontrol GmbH broadband dielectric spectrometer. Ionic liquid samples were placed in a parallel-plate sample cell equipped with stainless steel electrodes, maintaining a uniform electrode spacing of 3 μm. Measurements were conducted in the frequency range of 10^–2^–10^7^ Hz, and in the temperature range of 133–373 K. Conductivity data were collected every 10 K, with a stabilization time of 180 s at each temperature. The discussed conductivity values are those collected upon heating. An alternating voltage of 0.01 V was applied to minimize electrode polarization effects. The temperature was controlled with a nitrogen gas cryostat, ensuring a stability of ±0.1 K.

#### Raman Spectroscopy

Room-temperature Raman spectra were obtained using a Renishaw InVia Reflex Raman spectrophotometer equipped with an air-cooled CCD detector and a 785 nm diode laser as the excitation source. The laser power was set to 5% of its maximum value, which is 300 mW at the source. A grating with 1200 grooves per millimeter and a Leica objective of ×50 were used, yielding an approximate spectral resolution of 2 cm^–1^. Prior to analysis, the ionic liquid samples were sealed in NMR tubes, using PTFE sealing tape, inside a glovebox to prevent moisture uptake. Spectra were recorded over the range 100–4000 cm^–1^, with each measurement consisting of 10 accumulations at an acquisition time of 10 s per scan. Calibration of the instrument was carried out before each experiment using the first-order vibrational mode of a Si wafer calibrated at 520.6 cm^–1^. The collected Raman spectra were treated by excluding sharp signals coming from cosmic rays. Peak positions were determined using a multipeak fitting procedure based on a linear baseline and Voigt functions in the Igor Pro 9 software, with no constraints in width or intensity.

#### Infrared Spectroscopy

Infrared spectra were collected using an ALPHA II Bruker infrared spectrometer in the ATR (Attenuated Total Reflection) mode using a single-reflection diamond crystal. Measurements were conducted inside a glovebox to avoid interferences due to absorption of moisture. Spectra were acquired over the 200–4000 cm^–1^ spectral range with 128 accumulations, hence achieving a spectral resolution of 2 cm^–1^. The position of the N–H stretching mode was determined using a multipeak fitting procedure in Igor Pro 9, employing a linear baseline and Voigt functions, with no constraints in width or intensity.

#### Nuclear Magnetic Resonance Spectroscopy

Quantitative NMR data were collected using a Bruker Avance III HD 800 MHz spectrometer. The ionic liquid samples were loaded in 3 mm NMR tubes fitted with a capillary filled with D_2_O. The NMR tubes were filled and sealed inside a glovebox and spectra were acquired at 298 K. The pulse angle was set to 30°, the relaxation delay was set to 15 s, and 32 scans were collected.

Diffusometry NMR measurements were conducted on an AVANCE III HD Bruker NMR spectrometer operating at 14.1 T, equipped with a Diff30 probe and a 60 A gradient amplifier. Two 5 mm RF coil inserts were used, a ^1^H/^2^H double coil and a ^19^F single coil. Self-diffusion coefficients were determined at six temperatures between 293 and 343 K. Liquid samples were loaded into 5 mm NMR tubes and sealed inside a glovebox. A standard stimulated echo pulse sequence (diffste) was used at room temperature, whereas a double stimulated echo sequence (diffDste) was applied at high temperatures to eliminate artifacts caused by thermal convection. A 18 μs 90-degree pulse, 1 s acquisition time, 5 s recycling delay, 100 ms diffusion delay (Δ), and 1 ms gradient pulse duration (δ) were used. The maximum gradient strength (*g*) was varied in the range 100–1200 G/cm while keeping *k* constant, *k* being

1 $$k=(\delta \cdot \gamma \cdot g{)}^{2}\left(\Delta -\delta /3\right)$$

where δ is the gradient pulse duration, γ is the gyromagnetic ratio of the studied nucleus, *g* is the gradient strength, and Δ is the diffusion delay time. The gradient strengths were calibrated using reference samples with known self-diffusion coefficient values, i.e., the ^1^HDO trace signal in D_2_O reference sample and a hexafluorobenzene (C_6_F_6_) sample, for ^1^H and ^19^F respectively.^23^ In the analysis of the intensity attenuation, the average of all ^1^H NMR resonances, except for the N­(H) signal, was considered for the cations. The signal at −80 ppm in the ^19^F spectrum was used for the [TFSI] anion. The temperatures were calibrated to the chemical shift differences in pure methanol and pure ethanol glycol prior to and after the experiments.^24^

#### Electrochemical Analysis

The electrochemical stability windows were determined using the linear sweep voltammetry (LSV) method. The measurements were conducted in a 2032 coin cell, using stainless steel as the working electrode and metallic Li-foil (200 μm thickness, 35 mm diameter and purity ≥99.8%, Honjo Metals) as the combined counter and reference electrode (*A* = 0.95 cm^2^). A circular Solupor separator (ϕ = 11 mm) was soaked with 60 μL of the ionic liquid. All cells were assembled and hermetically sealed in an Ar-filled glovebox. The LSV experiments were carried out from −0.5 to 6 V vs Li/Li^+^ with a 0.5 mV/s scan rate. The cyclic voltammetry (CV) measurements were performed between 0 and 2 V vs Li/Li^+^ at various scan rates ranging from 0.1 to 5 mV/s. The cell was cycled three times, with an open-circuit voltage of 1 h between cycles.

## Results and Discussion

### Thermal Properties

The thermal properties of the protic ionic liquids were investigated using TGA and DSC methods, Figure 2. The TGA thermograms show that all three ionic liquids undergo decomposition in two successive steps, Figure 2a. However, the two ionic liquids [HBim]­[TFSI] and [HPhim]­[TFSI] show a slightly lower decomposition temperature (*T*_d_) (Table 1) compared to the reference [HEim]­[TFSI], reflecting a lower thermal stability. The thermal decomposition of protic ionic liquids generally begins with a reverse proton transfer from the cation to the anion, forming neutral species that evaporate more easily than ion pairs.^25^ Thus, the reduced thermal stability of the two investigated protic ionic liquids can be attributed to the nature of their N–H bond caused by substituting the ethyl group with a phenyl group. This substitution enhances the electron-withdrawing effect through the imidazolium ring, resulting in an increased acidity of the N–H bond, consequently lowering the thermal stability. These results are consistent with the work of Miran et al., who investigated a series of protic ionic liquids based on a strong base 1,8-diazabicyclo­[5,4,0]-7-ene (DBU) and Brønsted acids of varying strength. The authors reported that the decomposition temperature is higher for PILs with stronger N–H bonds, corresponding to weaker cation–anion interactions, than for those with weaker N–H bonds.^25^

**Figure 2:**
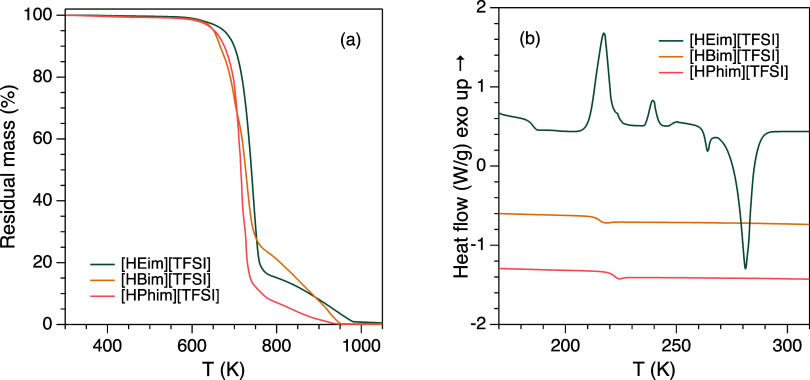
(a) TGA and (b) DSC thermograms of the neat protic ionic liquids.

**Table 1:** Decomposition Temperatures, Estimated from TGA, and Glass Transition Temperatures, Extracted from DSC (Second Heating Scan)

sample	*T*_d_ (K)	*T*_g_ (K)
[HEim][TFSI]	744	186
[HBim][TFSI]	728	214
[HPhim][TFSI]	717	219

The DSC data show a clear difference between the two studied protic ionic liquids and the reference [HEim]­[TFSI], Figure 2b. Contrarily to [HEim]­[TFSI] which shows both glass transition, cold crystallization and melting, [HPhim]­[TFSI] and [HBim]­[TFSI] are stronger glass-forming liquids, exhibiting glass transitions only. However, the glass transition temperatures (*T*_g_) of these two ionic liquids are significantly higher than that of the reference sample [HEim]­[TFSI], Table 1. This increase in *T*_g_ reflects stronger intermolecular interactions, which may result from the increased acidity of the cation N–H bond, in turn leading to enhanced hydrogen bonding and, hypothetically, favored proton exchange processes. Notably, an increase in *T*_g_ correlates to a decrease in *T*_d_, as illustrated in Figure S2.

### Intermolecular Interactions

The nature of intermolecular interactions was explored by Raman spectroscopy. Figures S3–S4 in the Supporting Information show the room temperature Raman spectra of both [HBim]­[TFSI] and [HPhim]­[TFSI]. The [TFSI] anion exhibits a strong and characteristic Raman band in the 740–750 cm^–1^ range, attributed to its expansion-contraction mode (ν_s_ S–N–S and ν_s_ CF_3_).^26^ The sensitivity of this mode to the strength of interactions experienced by the [TFSI] anion has been investigated earlier, by experimental and theoretical methods.^27,28^ For weakly coordinated [TFSI], this mode generally appears around 742–744 cm^–1^, while it shifts to higher wavenumbers (blue shift) in the presence of spectroscopically stronger interactions.^29–31^ The Raman spectra in Figure 3a, show that this vibration appears around 743 cm^–1^ for all studied samples, suggesting that the [TFSI] anion experiences spectroscopically weak interactions with its surrounding cations in all three ionic liquids. However, a peak fitting analysis of the 720–770 cm^–1^ range allowed an accurate determination of this peak position. A slight red shift is observed in the [HPhim]­[TFSI] and [HBim]­[TFSI] samples compared to [HEim]­[TFSI], Figure 3b. The observed red shift can be attributed to increased charge delocalization in the [HPhim] and [HBim] cations, compared to the case of [HEim], leading to weaker electrostatic interactions between the ions. Moreover, the S=O stretching mode observed around 1136 cm^–1^ (Figure 3c) is sensitive to H-bonding, since the N–H group on the cation is known to interact with the partially negatively charged oxygen atoms of the anion. Shifts in the position of the S=O peak reflect variations in the S=O bond length, which in turn relate to differences in the strength of cation–anion interactions. The position of this peak was hence determined by peak fitting the 1050–1200 cm^–1^ region, as shown in Figure S5. As shown in Figure 3d, the S=O stretching mode exhibits a red shift when moving from [HEim]­[TFSI] to [HBim]­[TFSI] and [HPhim]­[TFSI], reflecting an elongation of the S=O bond in [HBim]­[TFSI] and [HPhim]­[TFSI] relative to the reference sample, indicative of stronger cation–anion interactions.

**Figure 3:**
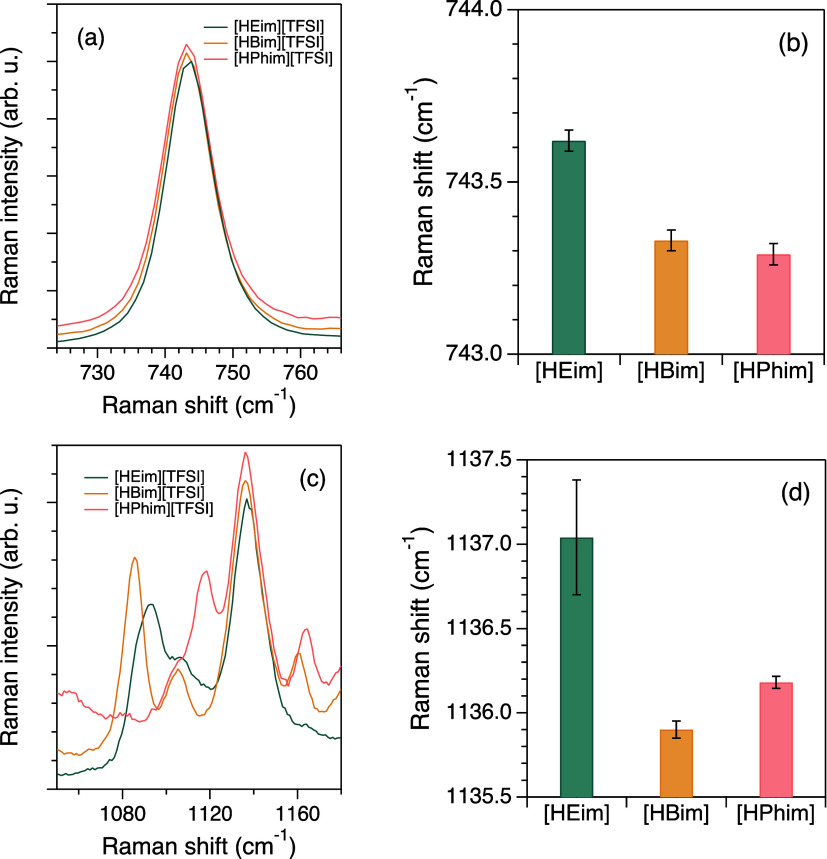
Room temperature Raman spectra of the protic ionic liquids in the 720–770 cm^–1^ (a) and the 1050–1180 cm^–1^ (c) ranges. The spectra are shown with a vertical offset for clarity. Raman shift of the [TFSI] sensitive mode (b) and of the S=O stretching mode (d) for the different ionic liquids’ cations. The error bars are extracted from the fitting.

Deeper insight into the nature of cation–anion interactions, particularly by means of hydrogen bonds, was gained through analysis of the N–H stretching mode in the infrared spectra. Figure 4a shows the infrared spectra of the three protic ionic liquids in the 2800–3600 cm^–1^ spectral range. The N–H stretching mode shows a broad peak around 3270 cm^–1^ for all three samples. A detailed peak fitting analysis of this region reveals a gradual red shift of the N–H stretching mode from [HEim]­[TFSI] to [HBim]­[TFSI], and then to [HPhim]­[TFSI], Figure 4b. An example of the applied peak fitting is shown in Figure S6. This red shift reflects the elongation of the N–H bond, in line with the increased bond acidity discussed above and indicative of stronger H-bonds between cations and anions.

**Figure 4:**
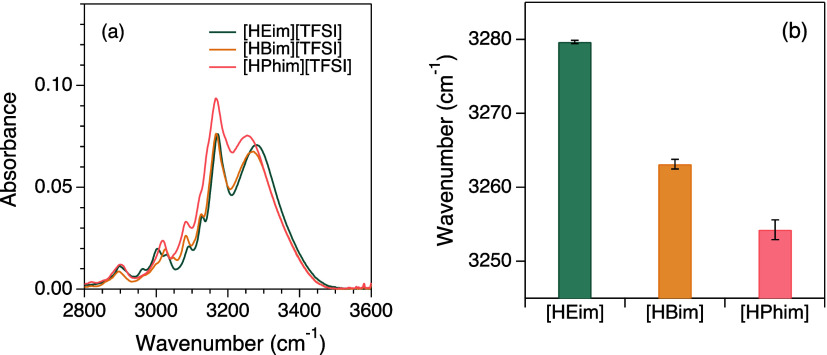
(a) Infrared spectra of the ionic liquids in the 2800–3600 cm^–1^ range. (b) The frequency of the N–H stretching mode as a function of the ionic liquids’ cation. The error bars are extracted from the fitting procedure.

NMR spectroscopy was used to further examine the nature of the cationic N–H bond. The ^1^H NMR spectra of the three ionic liquids are shown in Figure 5, while detailed peak assignments and integrals are provided in the Supporting Information, Figures S7–S9. It should be noted that the integrated area under the N­(H) signal corresponded to the expected stoichiometric value (1) in all three ionic liquids, indicating complete protonation of the reacting base. Figure 5 also shows that the N­(H) signals of [HBim]­[TFSI] and [HPhim]­[TFSI] appear at higher resonance frequencies compared to that of [HEim]­[TFSI]. A change in the ^1^H NMR chemical shift reflects a change in the electron density surrounding the proton. A decrease in electron density leads to deshielding and results in a shift toward higher resonance frequencies, typically due to nearby electronegative groups pulling electrons away from the hydrogen atoms. Hence, the more electron-withdrawing phenyl substituent on the imidazolium ring, replacing the ethyl, reduces the electron density around the N­(H) proton. This explains the observed shift of the N­(H) resonance. This is congruent with the other results, also suggesting stronger hydrogen bonding between cations and anions in the order [HPhim]­[TFSI] > [HBim]­[TFSI] > [HEim]­[TFSI].

**Figure 5:**
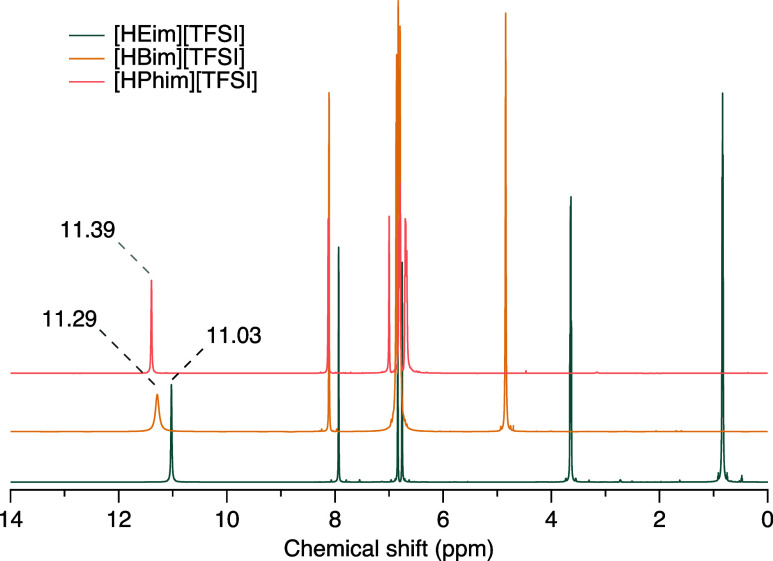
^1^H NMR spectra of the three protic ionic liquids, all collected at room temperature.

As a summary, the change in the cation structure impacts the nature of the N–H bond, which becomes more acidic in [HBim]­[TFSI] and [HPhim]­[TFSI] compared to [HEim]­[TFSI]. This results in a red shift of the N–H stretching mode (infrared) and a concomitant shift downfield of the N­(H) resonance (NMR). This correlation is visualized in Figure S10.

### Transport Properties

The strength of intermolecular interactions is known to significantly influence the macroscopic ionic conductivity of an ionic liquid. The Arrhenius plots of ionic conductivity are shown in Figure 6a,b, for the neat ionic liquids. [HEim]­[TFSI] shows the highest values, followed by [HBim]­[TFSI], while [HPhim]­[TFSI] exhibits the lowest conductivity. This trend is consistent with that of *T*_g_, where a higher *T*_g_ is expected to result in higher viscosity and lower ionic conductivity (Table S1). Moreover, for all ionic liquids, the ionic conductivity increases with increasing temperature. Note that abrupt changes in the ionic conductivity are observed (around 323 K for [HBim]­[TFSI] and around 313 K for [HPhim]­[TFSI]), although no first-order phase transitions were detected in the DSC thermograms at these temperatures (see Figure S11). This discrepancy can be attributed to either the different cooling rates used (20 K/min in DSC and 10 K/min in BDS) or to the fact that temperature is scanned continuously during DSC experiments whereas BDS measurements require an equilibration time at each selected temperature.

**Figure 6:**
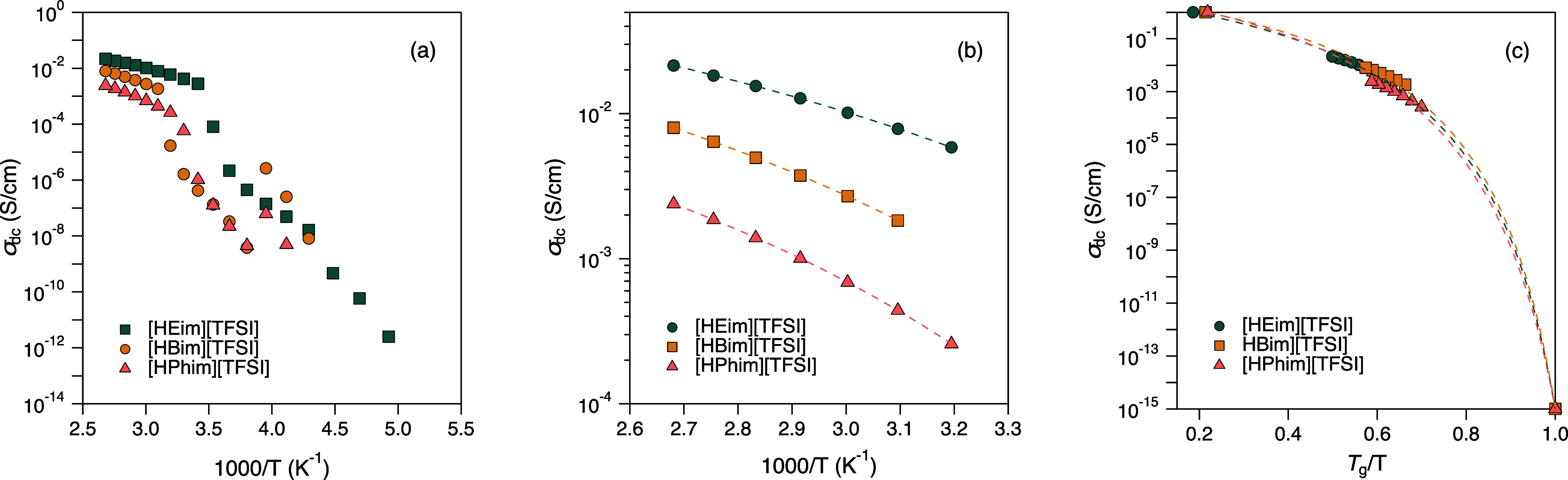
(a) Ionic conductivity of the three ionic liquid samples in the 203–373 K temperature range. (b) Zoom-in covering the 303–373 K temperature range. (c) *T*_g_-scaled Arrhenius plot (Angell’s plot) based on the experimental data shown in (b). The dashed lines are guides to the eyes.

The ionic conductivity values were also plotted on a *T*_g_-scaled Arrhenius plot (an Angell’s plot), normalizing the temperature scale to the glass transition temperature of each ionic liquid, Figure 6c. The *T*_g_ values used in this analysis are those obtained from DSC (see also Table 1). This representation allows the discussion of the ionic conductivity to be extended to the concept of fragility, a property of glass-forming systems that reflects how rapidly transport properties change at temperatures very close to the *T*_g_.^32^ In an Angell’s plot, fragile liquids exhibit a strong curvature, while strong liquids display a nearly linear behavior. In Figure 6c, two points have been added to the experimentally measured values, following the approach discussed in ref 32. That is, a point at *T* = *T*_g_ with a conductivity value of 10^–15^ S/cm, and a point at *T* = 1000 K with a conductivity value of 1 S/cm.^32,33^ This set of values is arbitrary but reflects a change in conductivity by 16 orders of magnitude, consistent with the hypothesized coupling to viscosity changes (where the relaxation time τ varies from 100 s to 10^–14^ s over the corresponding temperature range^34^). This approach enables a phenomenological fit over a broad temperature range, here showing that the ionic conductivities of the three ionic liquids fall onto very close curves. This indicates that their temperature-dependent conductivities evolve similarly relatively to *T*_g_. This observation suggests that, despite differences in cation structure and glass transition temperature, the transport properties of these ionic liquids show a similar temperature dependence once normalized to *T*_g_.

The conductivity data were then combined with density and viscosity (Table S1) to create a Walden plot and hence estimate the degree of ion association, Figure 7. In this plot, the diagonal dashed line represents the behavior of a 0.01 M KCl aqueous solution, where all ions are assumed to be fully dissociated and to move independently,^35^ while deviations from this reference can indicate stronger ion associations or decoupled ionic motion. The molar conductivities of all investigated ionic liquids fall below the ideal line, indicating partial ion association. Among them, [HPhim]­[TFSI] shows the largest deviation, suggesting a higher degree of ion pairing compared to [HBim]­[TFSI] and [HEim]­[TFSI]. This finding is consistent with its higher viscosity and higher glass transition temperature (*T*_g_), as previously discussed.

**Figure 7:**
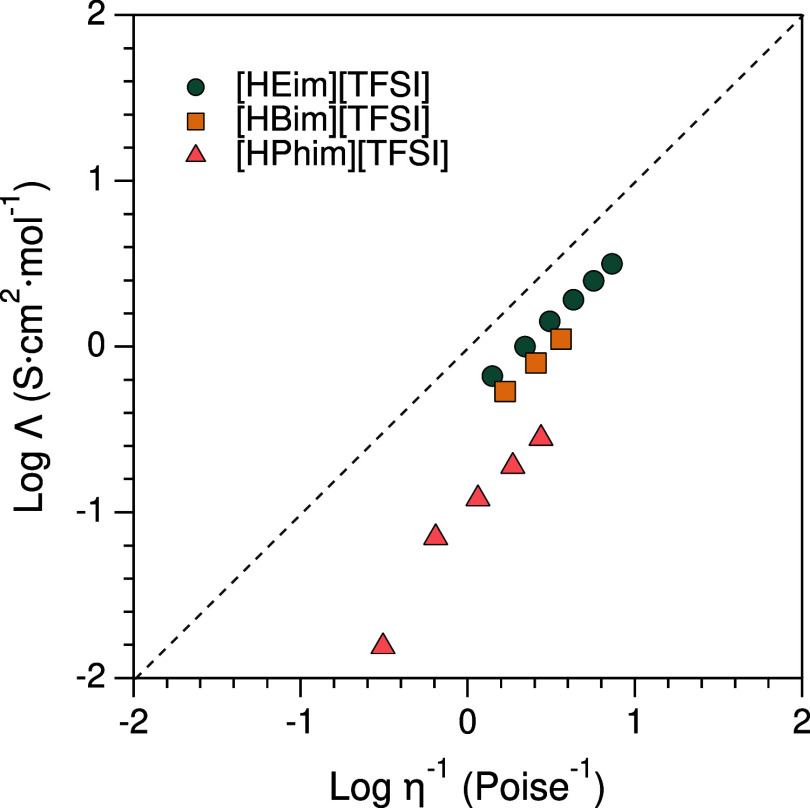
Walden plots for the protic ionic liquids [HEim]­[TFSI] (green circle), [HBim]­[TFSI] (yellow square) and [HPhim]­[TFSI] (pink triangle).

Furthermore, the transport properties of the studied ionic liquids were examined using NMR diffusometry, Figure 8a. The self-diffusion coefficients follow a similar trend to that of the ionic conductivity, with [HPhim]­[TFSI] and [HBim]­[TFSI] exhibiting lower diffusivity than [HEim]­[TFSI]. In [HEim]­[TFSI], the cations show higher diffusivity than the anions, consistent with previously reported studies. However, for [HBim]­[TFSI] and [HPhim]­[TFSI], the cation and anion diffusivities are nearly equal, which may be attributed to strong ion–ion interactions, suggesting their diffusion as ion pairs. The ^1^H and ^19^F self-diffusion coefficient values are summarized in Table S2.

**Figure 8:**
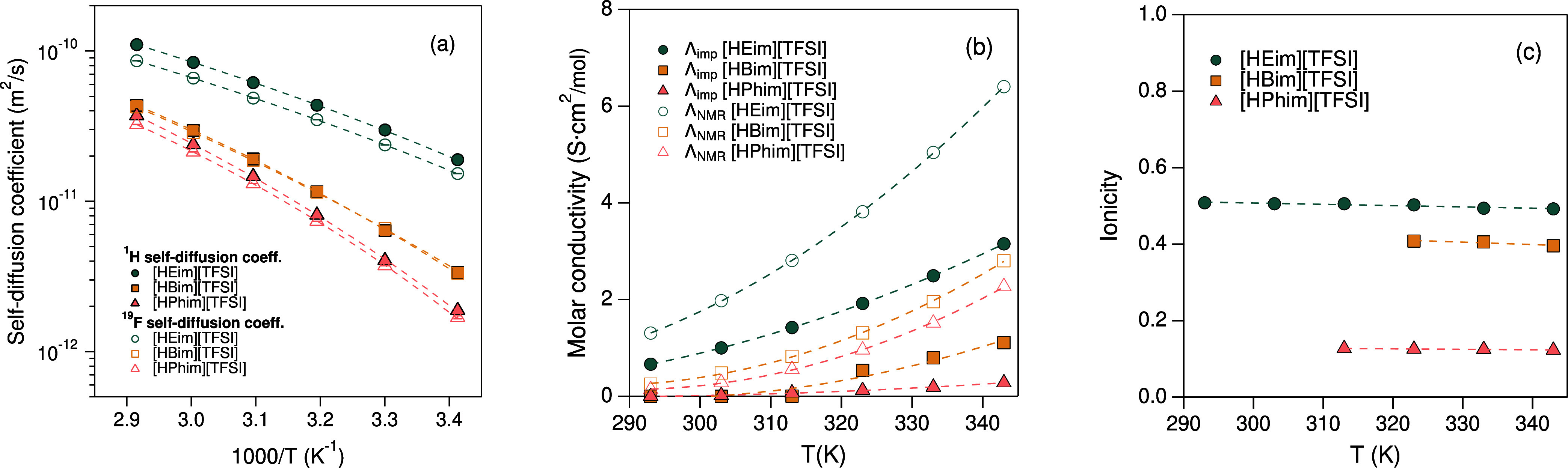
(a) ^1^H and ^19^F self-diffusion coefficients measured in the 293–343 K range for the three protic ionic liquids. The dashed lines are VFT fits. (b) Molar conductivities obtained from conductivity (filled symbols) and NMR (open symbols) measurements. (c) Values of ionicity as a function of temperature.

Another approach to assess the degree of ion association is by calculating their ionicity, as expressed in eq 2.^36–38^ The ionicity is defined as the ratio between the molar conductivity measured experimentally (eq 3) and the molar conductivity estimated from diffusion NMR data using the Nernst–Einstein relation (eq 4).

2 $$\mathrm{ionicity}=\frac{\Lambda_{\mathrm{imp}}}{\Lambda_{\mathrm{NMR}}}$$

3 $$\Lambda_{\mathrm{imp}}=\frac{\sigma_{\mathrm{dc}}\cdot M_{\mathrm{IL}}}{\rho }$$

σ_dc_ is the ionic conductivity, *M*_IL_ is the molar mass of the ionic liquid and ρ is the density.

4 $$\Lambda_{\mathrm{NMR}}=\frac{(D_{1_{H}}+D_{19_{F}})\cdot F^{2}}{R\cdot T}$$

*F* is the Faraday constant, *R* is the universal gas constant and *D*_*x*_ is the self-diffusion coefficient value of the *x* nucleus.

Figure 8b shows that Λ_imp_ is smaller than Λ_NMR_ for all three ionic liquids. This suggests faster short-range ion diffusion (probed by NMR) compared to long-range motion (probed by BDS), possibly caused by aggregates formation influencing long-range behavior. Moreover, the ionicity decreases drastically from [HEim]­[TFSI] to [HPhim]­[TFSI], the latter having an ionicity value close to 0.1. This observation highlights the fact that in [HPhim]­[TFSI], the ions move to a higher degree as ion pairs, which in turn is rationalized by the more acidic N–H bond, hence stronger cation–anion interactions.

### Electrochemical Analysis

Determining the electrochemical stability window is essential to evaluate the suitability of ionic liquids for energy storage applications, since a sufficiently wide stability is beneficial for practical use. The voltammograms of the three ionic liquids recorded between 0 and 6 V are shown in Figure 9a. The cathodic region, below 0 V, was not recorded as lithium plating was observed. Among the studied samples, [HEim]­[TFSI] shows the lowest anodic stability, with the oxidation reaction starting at ∼2.2 V. [HBim]­[TFSI] remains stable up to about 3.4 V, while [HPhim]­[TFSI] shows the highest anodic stability, up to 4.2 V. The latter high value is likely due to stronger cation–anion interactions that hinder oxidation of the [TFSI] anion. It should be mentioned that protic ionic liquids exhibit slightly narrower electrochemical stability windows than their aprotic counterparts, as reported in the literature for a series of imidazolium-based protic ionic liquids containing [TFSI].^39^

**Figure 9:**
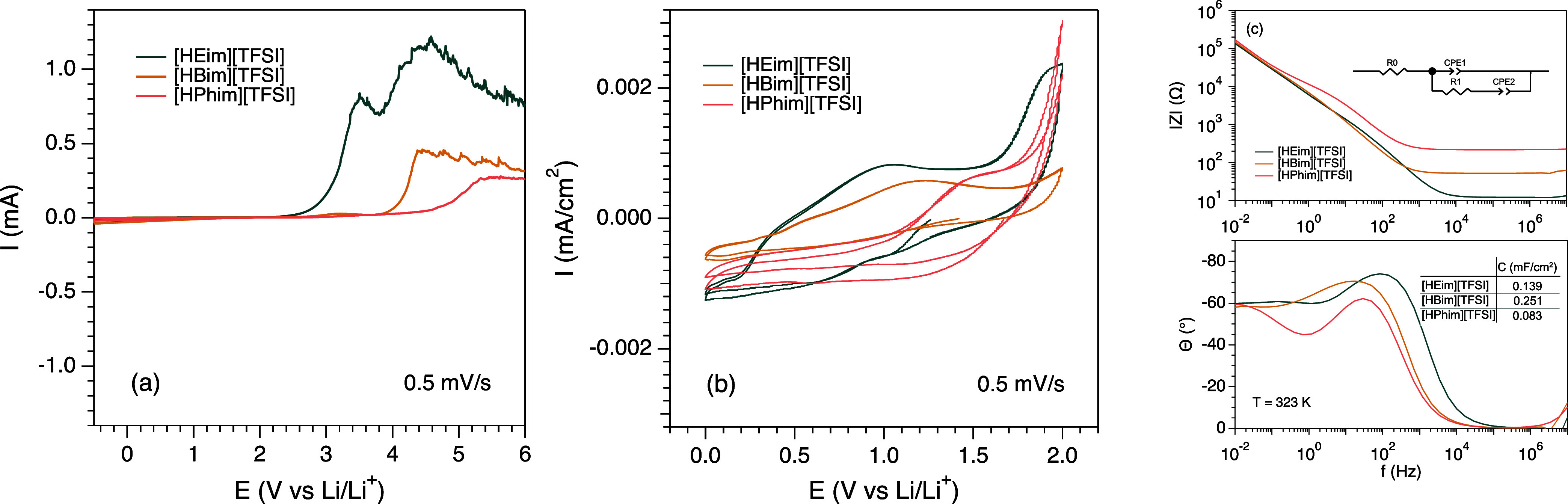
(a) Linear sweep voltammetry of the ionic liquids from 0 to 6 V vs Li/Li^+^ at a scan rate of 0.5 mV/s. (b) Cyclic voltammetric curves at a 0.5 mV/s scan rate. (c) Impedance modulus and Theta as a function of the frequency for the three ionic liquids. The insets show the equivalent circuit used to model the impedance data recorded and the low frequency capacitance values for the studied PILs.

Cyclic voltammograms (CVs) were further recorded in a narrower potential range between 0 and 2 V. Each measurement started from the open circuit potential (approximately 2 V). The CVs of the three ionic liquids at 0.5 mV/s scan rate are shown in Figure 9b. The measured current densities in this region are about 3 orders of magnitude lower than those observed over the wider potential window (0–6 V), indicating minimal electrochemical activity. However, the three ionic liquids exhibit distinct electrochemical behaviors. To gain further insight, CVs were recorded at different scan rates ranging from 0.1 to 5 mV/s (Figure S13). After normalization to the scan rate (Figure S14), the voltammograms for each sample do not overlap, suggesting the presence of both interfacial processes, i.e., double-layer capacitance, and Faradaic reactions.

Electrochemical impedance data, at 323 K, were then used to investigate the capacitive properties of the ionic liquids. The frequency dependence of the impedance modulus (|*Z*|), shown in Figure 9c, reveal a quasi-linear behavior below 10^4^ Hz and a frequency-independent plateau above 10^4^ Hz. The high-frequency plateau corresponds to the bulk resistance of the ionic liquids, whereas the low-frequency slope is associated with processes occurring at the electrode/electrolyte interface. The phase angle as a function of frequency is also presented in Figure 9c (lower panel). The frequency dependence of |*Z*| was analyzed using equivalent circuit modeling, where the equivalent circuit describing the studied ionic liquids included a resistance R0 and a constant phase element CPE1 in parallel with a resistance R1 and a CPE2. The capacitance was estimated using the following relation:^40^

5 $$C=Q^{1/\alpha }\cdot R_{\mathrm{sol}}^{\left(1-\alpha \right)/\alpha }$$

where *Q* and α are parameters related to the constant phase element (CPE), *Q* is a frequency dependent capacitance (F·s^(α–1)^) and α is a dispersion factor, *C* is the effective capacitance (F) and *R*_sol_ is the solution resistance (Ω). Taking into account that the total active area during the EIS experiments is 0.0314 cm^2^, the specific double-layer capacitance of the three samples, at 323 K, could be determined. Among the studied samples, [HBim]­[TFSI] exhibits the highest specific capacitance (0.251 mF/cm^2^). Although it does not have the widest electrochemical stability window, its higher capacitance may be a consequence of a more efficient ion packing at the electrode interface.

## Conclusion

In this study, two newly commercialized protic ionic liquids, [HBim]­[TFSI] and [HPhim]­[TFSI], were thouroughly characterized, with focus on phase behavior, intermolecular interactions, and transport properties. Found properties are benchmarked to those of the more widely used ionic liquid [HEim]­[TFSI]. A red shift of the [TFSI]-characteristic Raman mode, a red shift of the N–H stretching mode in infrared spectra, and a shift downfield of the N­(H) resonance in ^1^H NMR were observed, reflecting an increased acidity of the N–H bond in the imidazolium cation in the new protic ionic liquids, compared to [HEim]­[TFSI]. This increase in acidity is attributed to the electron-withdrawing effect caused by the substitution of the ethyl group with the more electronegative phenyl group.

Lower thermal stability (*T*_d_), higher glass transition temperatures, and consequently reduced ionic conductivity and self-diffusion coefficients were observed in these new protic ionic liquids. These findings were interpreted as an evidence of stronger interionic interactions resulting from the increased acidity of the N–H bond. Although high viscosity and low ionic conductivity were observed, limiting their performance as electrolytes for ion conduction, the new ionic liquids might have promising potential for application in proton exchange membranes or catalysis. These possibilities remain to be further explored in future studies.

## Supplementary Material



## Notes

Additional data obtained from DSC, TGA, Raman spectroscopy, ^1^H NMR, FT-IR, viscosity and density measurements, NMR diffusometry, and electrochemical data analysis (PDF)
