# Supplementary material for: Structure–Property Correlation in Phenyl N‑Substituted Imidazolium Protic Ionic Liquids
Source: J Phys Chem B. 2026 Jun 23;130(27):6871–9. doi: 10.1021/acs.jpcb.6c01792 (PMC13359107; doi:10.1021/acs.jpcb.6c01792)
Supplement: Supplementary file 1 [file jp6c01792_si_001.pdf]

Supporting Information: Structure-Property Correlation in Phenyl N-Substituted Imidazolium Protic Ionic Liquids

Nicole Abdou,*,† Elisabet Ahlberg,‡ and Anna Martinelli*,†

†*Department of Chemistry and Chemical Engineering, Chalmers University of Technology, SE-412 96 Gothenburg, Sweden*

‡*Department of Chemistry and Molecular Biology, University of Gothenburg, SE-413 90 Gothenburg, Sweden*

E-mail: nicole.abdou@chalmers.se; anna.martinelli@chalmers.se

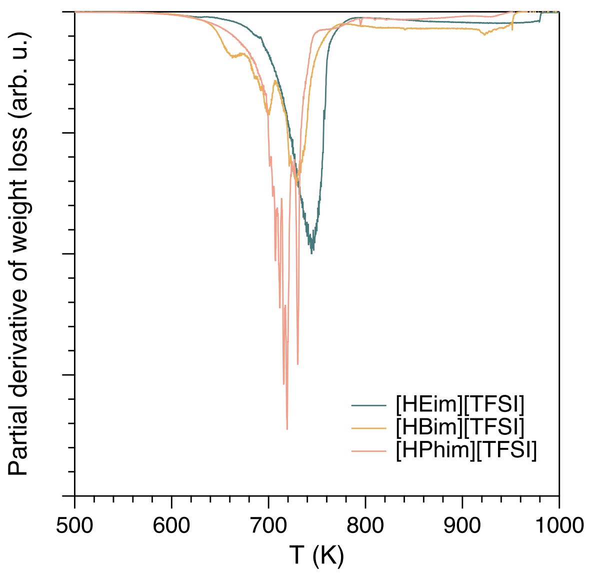

Figure S1: Partial derivative of weight loss (arb. u.) estimated for all three samples and used to determine the decomposition temperatures (T_d).

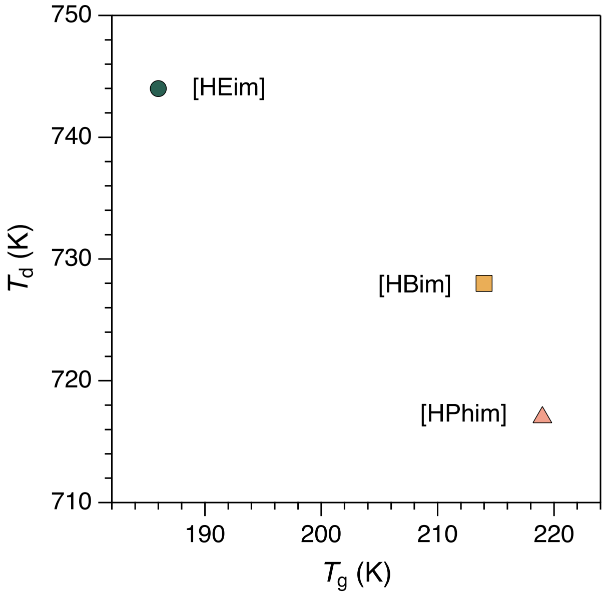

Figure S2: Decomposition temperature (T_d) as a function of the glass transition temperature (T_g) for all three ionic liquids.

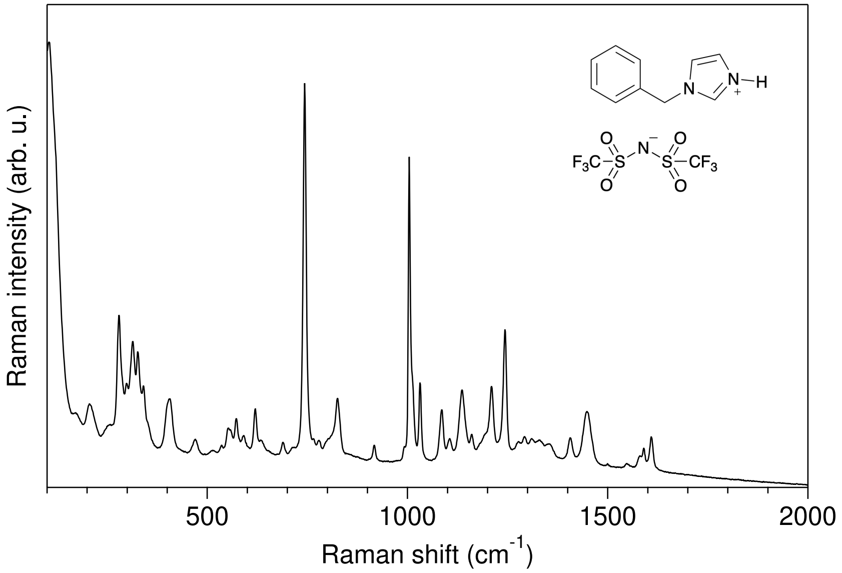

Figure S3: Raman spectrum of the protic ionic liquid [HBim][TFSI].

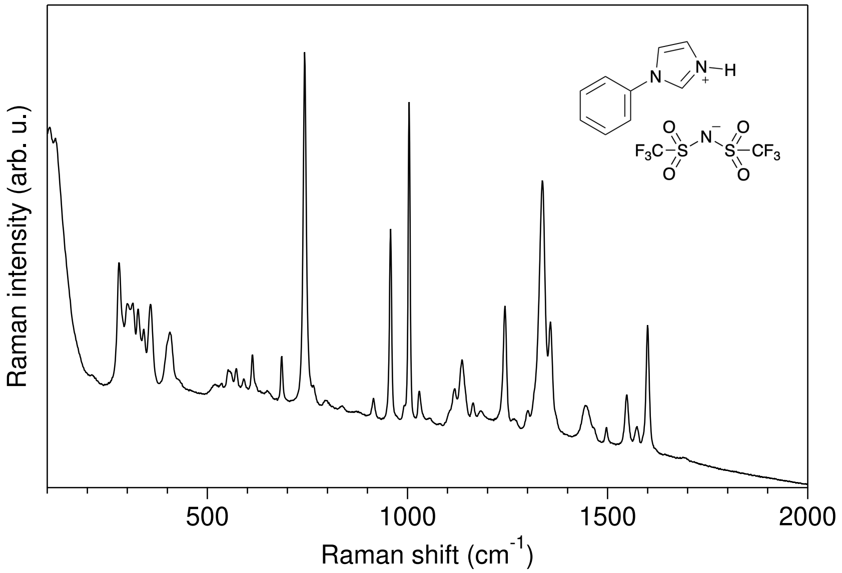

Figure S4: Raman spectrum of the protic ionic liquid [HPhim][TFSI].

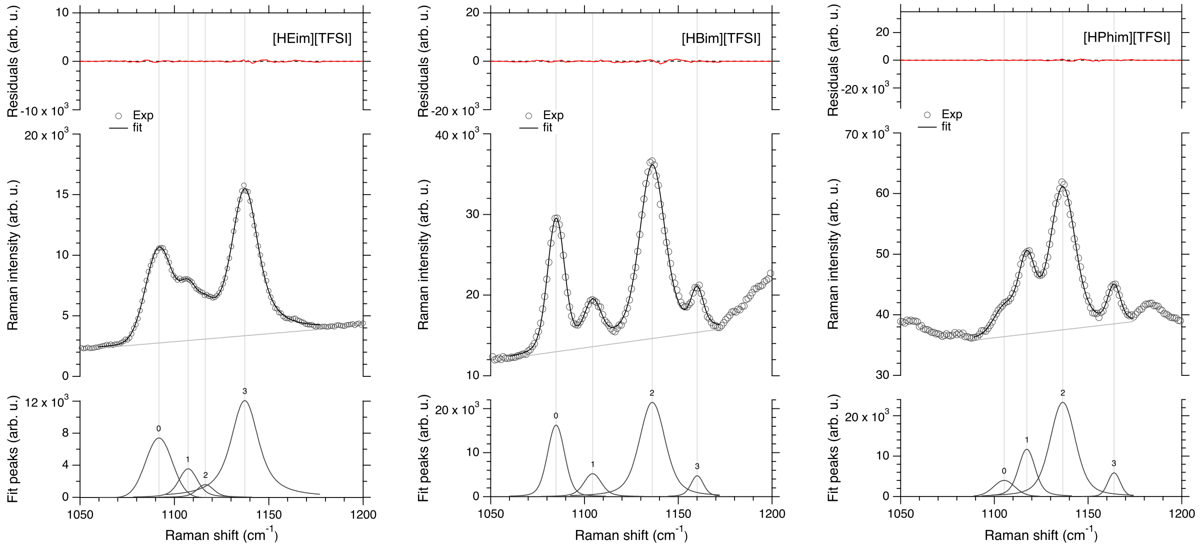

Figure S5: Peak fitting procedure and results for the Raman spectra of the three studied protic ionic liquids in the 1050 – 1200 cm^{-1} spectral range.

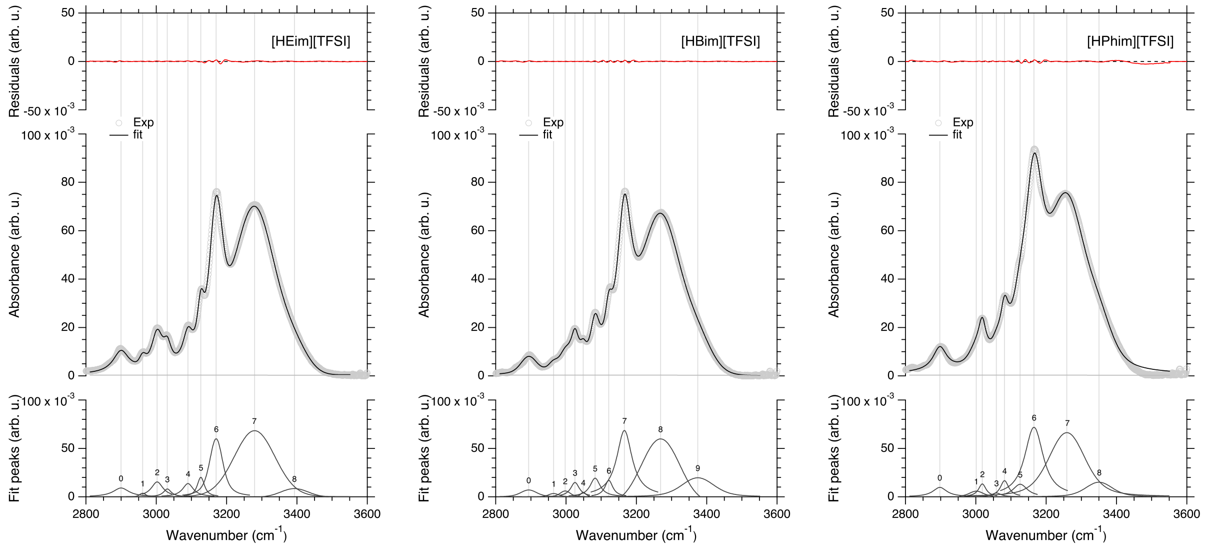

Figure S6: Peak fitting procedure and results for the Infrared spectra of the three studied protic ionic liquids in the 2800 – 3600 cm^{-1} spectral range.

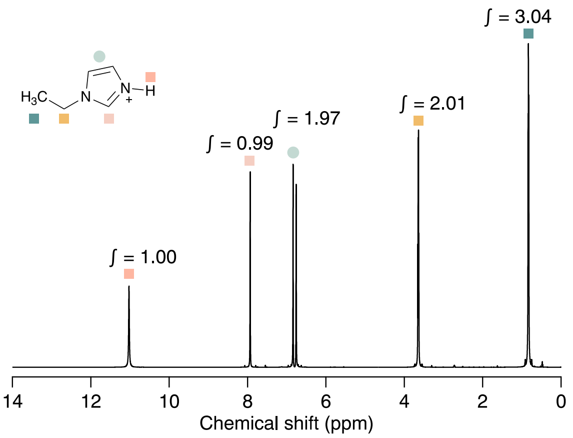

Figure S7: ^{1}H NMR spectrum of the protic ionic liquid [HEim][TFSI].

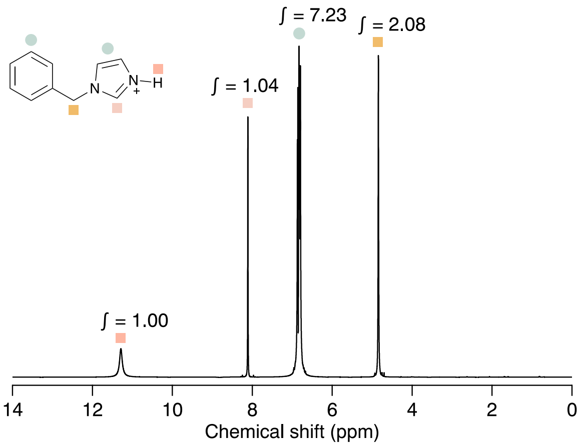

Figure S8: ^{1}H NMR spectrum of the protic ionic liquid [HBim][TFSI].

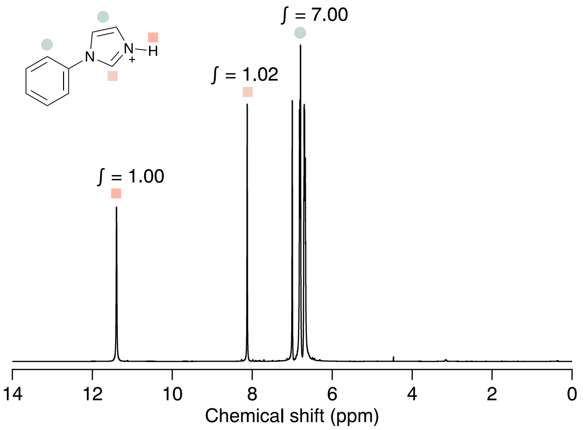

Figure S9: ^{1}H NMR spectrum of the protic ionic liquid [HPhim][TFSI].

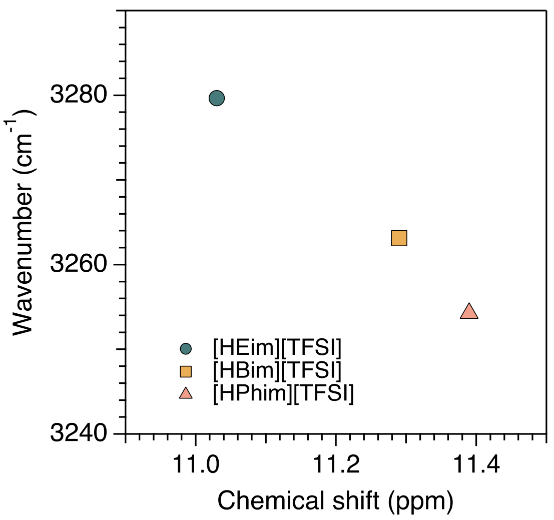

Figure S10: Dependence of the FT-IR wavenumber on the ^{1}H chemical shift for all three protic ionic liquids.

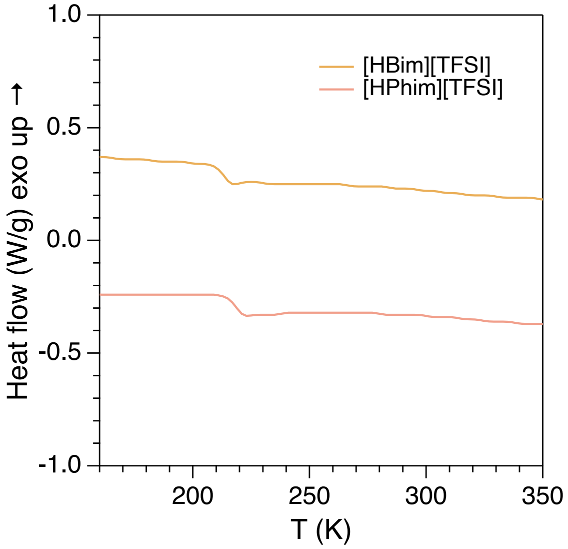

Figure S11: Extended-range DSC thermograms of the studied protic ionic liquids.

Table S1: Density and viscosity values, collected at different temperatures, for all three protic ionic liquids.

Sample	T (K)	Density (g/cm^3)	Viscosity (mPa·s)
[HEim][TFSI]	293	1.57	71.18
	303	1.57	45.47
	313	1.56	32.25
	323	1.55	23.27
	333	1.54	17.67
	343	1.52	13.70
[HBim][TFSI]	293	1.53	359.14
	303	1.52	176.86
	313	1.51	98.04
	323	1.50	59.67
	333	1.49	39.09
	343	1.48	27.63
[HPhim][TFSI]	293	1.58	776.63
	303	1.57	321.18
	313	1.56	155.53
	323	1.55	86.81
	333	1.54	53.80
	343	1.53	36.44

Table S2: Cation (^{1}H) and anion (^{19}F) self-diffusion coefficients measured at different temperatures ranging from 293 K to 343 K. The errors from the fitting are smaller than the represented figures.

Sample	T (K)	$D_{^1\mathrm{H}}$ 10^{-11} (m^2/s)	$D_{^{19}\mathrm{F}}$ 10^{-11} (m^2/s)
[HEim][TFSI]	293	1.89	1.53
	303	2.98	2.37
	313	4.36	3.49
	323	6.15	4.86
	333	8.40	6.60
	343	11.02	8.60
[HBim][TFSI]	293	0.33	0.33
	303	0.64	0.66
	313	1.16	1.15
	323	1.91	1.87
	333	2.96	2.86
	343	4.35	4.24
[HPhim][TFSI]	293	0.19	0.17
	303	0.40	0.37
	313	0.81	0.74
	323	1.46	1.31
	333	2.38	2.13
	343	3.72	3.24

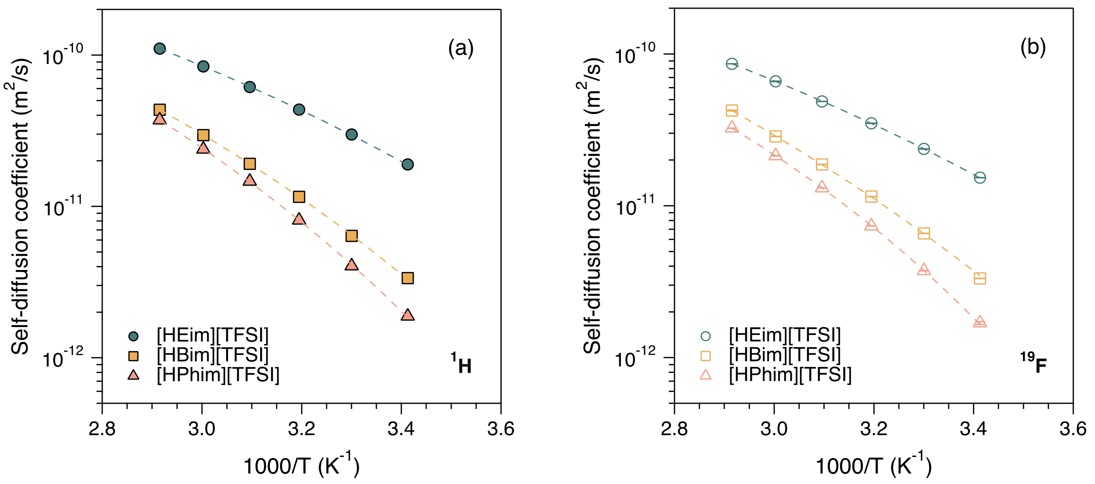

Figure S12: (a) Cation (^{1}H) and (b) anion (^{19}F) self-diffusion coefficients measured in the 293 – 343 K range for the three ionic liquids. The dashed lines are guides to the eyes.

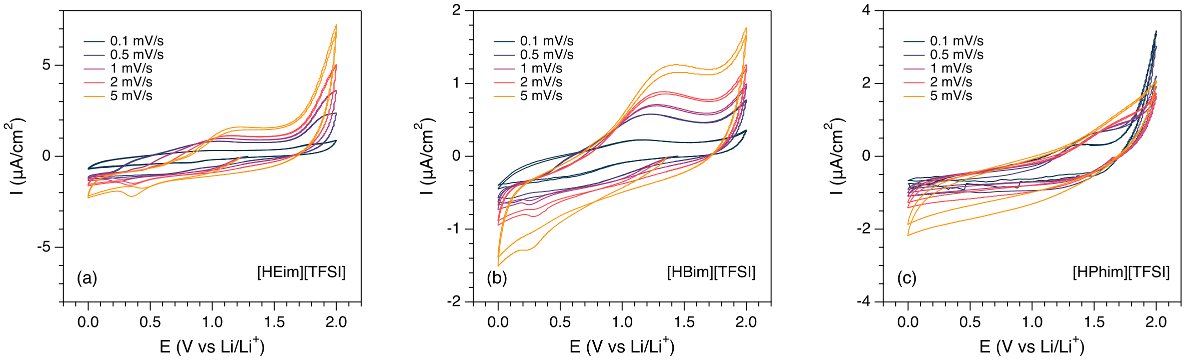

Figure S13: Cyclic voltammograms recorded at various scan rates for [HEim][TFSI] (a), [HBim][TFSI] (b) and [HPhim][TFSI] (c).

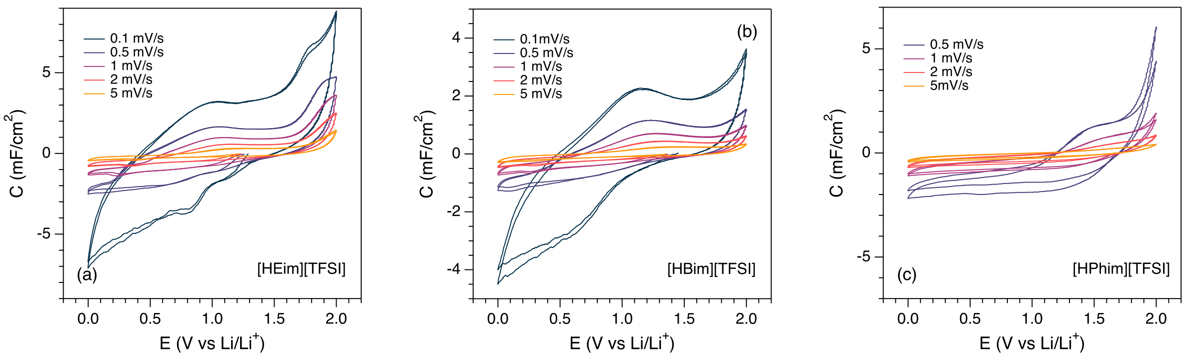

Figure S14: Double layer capacitance as a function of the potential (vs Li/Li$^+$) at different scan rates for [HEim][TFSI] (a), [HBim][TFSI] (b) and [HPhim][TFSI] (c). These voltammograms show that the predominant process is Faradaic, while the capacitive contribution is negligible.
